# Minimal information: an urgent need to assess the functional reliability of recombinant proteins used in biological experiments

**DOI:** 10.1186/1475-2859-7-20

**Published:** 2008-07-23

**Authors:** Ario de Marco

**Affiliations:** 1 COGENTECH, via Adamello 16, 20139, Milano, Italy

## Abstract

Structural characterization of proteins used in biological experiments is largely neglected. In most publications, the information available is totally insufficient to judge the functionality of the proteins used and, therefore, the significance of identified protein-protein interactions (was the interaction specific or due to unspecific binding of misfolded protein regions?) or reliability of kinetic and thermodynamic data (how much protein was in its native form?). As a consequence, the results of single experiments might not only become questionable, but the whole reliability of systems biology, built on these fundaments, would be weakened.

The introduction of Minimal Information concerning purified proteins to add as metadata to the main body of a manuscript would render straightforward the assessment of their functional and structural qualities and, consequently, of results obtained using these proteins. Furthermore, accepted standards for protein annotation would simplify data comparison and exchange. This article has been envisaged as a proposal for aggregating scientists who share the opinion that the scientific community needs a platform for Minimum Information for Protein Functionality Evaluation (MIPFE).

## Introduction

The introduction of standards for reporting experimental conditions and public access annotation of Minimal Information (MI) enables the development of homogeneous formats for data comparison and storage, and results in simplified data analysis and improved reproducibility. Research and industrial labs can rationalize their work and avoid losing information and know-how by storing data and protocols in homogeneous formats, objectively annotated and easily understandable. As a consequence, the results are accessible for control, further analyses and sharing, avoiding loss of competences once the operator has left. Furthermore, development of software and equipment is stimulated by having clearly defined standards. Scientific editors and funding agencies also profit from an established repository that simplifies data access, comparison, verification and exchange [1]. In summary, standardization increases the global value of results, as already described in detail [1-3]. The research communities operating in proteomics, microarray, and molecular interactions have already progressed in organizing their work through, for instance, the Proteomic Standard Initiative [4]. The resulting guidelines were incorporated in platforms like MIAPE, MIAME, MIMIx [3], MISFISHIE or MIGS aimed at the complete disclosure of methodologies used [5,6] and at description of both the data generated and their annotation. Conformation to these standards is already compulsory for publishing in several top journals [7,8]. An increasing number of projects in different bioscience fields is now organized under the umbrella of a central register for guidelines, the Minimal Information for Biological and Biomedical Investigation (MIBBI, see Availability and requirements section for URL), and another interesting approach is represented by interactive models as Human Proteinpedia (see Availability and requirements section for URL) and WikiProteins [9,10].

Probably, it is time to apply analogous standardized methods also to the field of protein, monoclonal, and recombinant antibody production, following as a model what has already been proposed and implemented so far [2-4,11].

## Time for MIPFE?

As mentioned in previous publications dealing with data collection standardization, there are general reasons for such choices, and all the parts will gain from producing and storing "transparent" data [1,3,12,13].

A specific reason for having MI in protein production technology is the fact that protein production is a relatively accessible technique, largely performed by non-specialists. As a consequence, there is often a superficial approach in dealing with the subject and a general underestimation of the critical control experiments and quality standards. General biology journals usually do not ask for proof that proteins used in experiments were monodispersed and natively folded, namely it is not possible to evaluate the congruence of published data, since information concerning the structure and functionality of the experimental material is not usually reported. This situation leads to ambiguous and contradictory results, and raises the necessity to identify a suitable and largely accepted laboratory information management system for protein production and characterization. The aim of this effort would be not to emphasize demanding analyses, but to help in finding rational and reproducible operative conditions, by offering standardized guidelines for procedures and rigorous annotations. An ideal Minimum Information for Protein Functionality Evaluation (MIPFE) platform to handle and characterize proteins would be appreciated by non-specialists as well as by biochemists and crystallographers. Furthermore, the introduction of defined and universal protein quality standards will be beneficial for the validation of the data used as "constitutive elements" in other MI platforms, like MIMIx and MIRIAM, which use *bona fide *publicly accessible results [14].

The three basic components of such a platform should include MI specifications, data formats, and controlled vocabulary [2]. The Ontology for Biomedical Investigations – OBI (see Availability and requirements section for URL) is the ongoing project aimed at providing the scientific community with appropriate terminology, and would be the reference.

Data formats are the units for information transmission, and their choice plays a crucial role in optimizing data sharing. The danger of fragmentation among different platforms has been claimed [1], whilst the maximal exploitation of research data will be possible only in a context in which data structures are harmonized. The Functional Genomics Experiment – FuGE (see Availability and requirements section for URL) Project could be considered as a reference model since it was specifically created for providing suitable Extensible Markup Language (XML) formats for MI initiatives [15]. Future alternatives, such as the mzML format that is still under development [8], would be considered.

Nevertheless, after having identified possible solutions for data formats and vocabulary, the most difficult questions for the community remain unanswered: what is Minimal Information? How do we fix the standards for unambiguous material description? There is a lot to learn from past experience.

## Minimal Information

It has been proposed to create three levels of information: what must be annotated, what should be accessible, and what is optional to insert [13]. Minimal information means that a set of metadata, sufficient to evaluate the data reliability, must be annotated, selecting unambiguous definitions. In other words, MI will request only compulsory data that are the minimal requirements for quality evaluation. Nevertheless, the same format could also host advanced information (for instance, the kind of information that now is boxed into manuscript supplementary data), and annotations mostly intended for internal use (lab archive of material and specific details for lab praxis optimization). However, these supplementary data will also be annotated using a standardized structure, with the aim of simplifying further retrieval and analyses avoiding loss of technical intelligence. The main advantage would be having data and metadata archived in an easily accessible form [15].

An example of Minimal Information on material and experimental condition description could correspond to the requests summarized in Table 1. A proposal of Minimal Information for functional/structural quality evaluation of specific protein manipulation is shown in Table 2. Protocols for standardized performances and general guidelines of good lab praxis would be also considered for discussion, with the idea of finding a consensus aimed at more uniform experimental conditions (Table 3).

**Table 1:** Material information sheet. The MI concerning the material preparation and characterization is reported

Construct information	Accession number; partial sequence?; mutations?; fusions to tags?; rec. Abs: format (Fab, scFv, VHH)
Vector information	Vector type and map; cloning sites; resistance; linkers; protease cleavage sites
Hybridoma clone	Identification; tests in ELISA, IP, WB, IHC
Chromatographic steps	IMAC (column1, buffer 1); Desalting (column 2, buffer 2); .....
Tag cleavage	Buffer; cleavage conditions
Protein storage conditions	Buffer; temperature; aliquots;
Re-folding	Buffer; strategy, glycosylation
Functionality	Enzymatic assay; IP; ELISA; IHC; SPR; ITC; Oxidation status (SH/S-S), Appearance, Stability
Purification parameters	Yields; Specific activity; K_D_; Aggregation index; multimerisation, specific activity
Purity	host cells proteins, nucleic acids, lipids, carbohydrates, process added chemicals

**Table 2:** Experimental information check list. MI for each experiment is in bold, optional information is in italics.

**Experiment type**	**Analyses to perform for** **evaluating proteins before** ** starting the experiments**	**Protocol references (from a list** **of accepted standard protocols** **for each experiment type that** ** guarantee output congruency)**	**Equipments** **(define minimal requisites of** **the devices in terms of** **quality, maintenance,** ** controls)**
Protein pull-down	**SDS PAGE; AI; ***SEC; DLS; CD far UV; CD near UV; FT-IR; NMR;*	SDS: protocol 1 (or protocol 2,..) AI protocol 1	SDS: Hoefer minigel.... AI: Spectrofluorimeter Jasco ..... SEC: column xy and FPLC model z.....
Monoclonal Ab purification	**SDS PAGE; AI; ***SEC; DLS; CD far UV; CD near UV;*		
Protein purification	**SDS PAGE; AI; SEC;***DLS; ***CD far UV**; *CD near UV; FT-IR; NMR;*		
SPR			
ITC			
ELISA			
Enzymatic assay			
Protein refolding			
Immunoprecipitation			
Immunofluorescence			
Immunohistochemistry			
Proximity ligation			

The corresponding pictures (SDS-gels, chromatographic profiles, fluorimeter spectra,.....) would be embedded in the metadata form.

*Abbreviations*: Aggregation Index (AI) (16), Size Exclusion Chromatography (SEC), Dynamic Light Scattering (DLS), Circular Dichroism (CD), Fourier-Transform InfraRed (FT-IR), Nuclear Magnetic Resonance (NMR).

**Table 3:** General guidelines for good lab praxis.

Clone only in expression vectors that allow cleavage of the tag after purification. **Advantage**: any side effect due to the tag can be evaluated	
Cleave the tag before using the target protein in interaction experiments. **Advantage: **unspecific interactions can be limited	
Prepare mono-use aliquots of the purified protein. **Advantage: **reproducibility of the experiment is improved	
Perform an experiment to evaluate protein stability after incubation on ice or at room temperature at different times; stability for freeze/thaw, if applicable. **Advantage: **experiment design is optimized	

## Towards clear and consistent standards

It is difficult to set exhaustive MI checklists and both engagement and acceptance from the scientific community are critical for the successful adoption of standards that will allow: a) evaluation of experimental consistency, and b) straightforward data exchange to get the most out of produced results [1,13,15]. Consequently, MIPFE platform, as most of the already existing similar initiatives, should be thought as a flexible tool. A preliminary draft expressing clear purposes and contents (for instance, a document extending the annotations reported in Tables 1 to 3) would be made accessible, ideally on an electronic discussion format, and public criticism and feed-back will be reviewed and eventually integrated. It has been underlined that premature adoption of formalized standards will result in the successive coexistence of original and more mature versions [13]. The criticism of producing annotations that are difficult to compare has been already expressed for MIAME [13]. Such a confusing situation would compromise the aim of the initiative since it would generate metadata in different and contrasting formats, preventing easy data sharing. Therefore, fixed, clear, and unambiguous standards should be defined and implemented only after comprehensive vetting by the community to avoid nebulous reporting consistency allowed by subjective interpretations of the requested detail level. Platform simplicity would be accomplished for avoiding that annotations could represent a burden for the scientist work.

## Lobbing for MIPFE

Accurate (new) analyses may represent a practical obstacle for some groups, although data reliability would have priority over technical limitations of single individuals. It could be objected that microarray and proteomic communities already decided that a further effort from scientists was necessary for improving the value of generated data.

Centralized facilities, specialized in protein and monoclonal antibody production, have been implemented in most of the larger research institutes for providing internal services. MIPFE could offer a great opportunity for their growth in terms of reliability and evaluation of results. Dealing with a large number of technically demanding but repetitive activities, facilities can afford the commitment to develop standardized quality control analyses with increasing levels of sophistication that could be difficult to achieve in non-specialized labs. Moreover, facilities could directly use MIPFE standard forms to annotate their work output. These forms will have the double advantage of certifying the activity towards customers and being already MI metadata annotated in a form directly usable by the community at large (editors, industry, funding agencies, researchers).

Journals should be contacted to discuss the implementation of electronic accessible forms and asked to request standard compliance as a condition for publication. Notably, this already happens for compulsory submission of DNA sequences to data repositories [1] and in the case of proteomics [7,8]. The main limitation is the lack of centralized repository data-bank, a general issue for any MI initiative, and journal websites would have to supply one for metadata storage. Fortunately, funding agencies become more and more sensitive to the MI initiatives [1,3] in the light of recognition that such platforms are crucial for efficient data dissemination and they will probably provide the necessary support in the next future. Furthermore, publicly supported initiatives, like Addgene (see Availability and requirements section for URL), may help in simplifying storage and availability of material used in the experiments.

The integration of MIPFE in one of the already pre-existing platforms would be investigated in the effort to avoid overlap among platforms.

## Conclusion

When MIAPE [3] was introduced, the authors underlined the necessity to contextualize data with "metadata". For instance, the statement that "the sequence corresponding to amino-acids 197 to 259 of NPM interacts with the N-terminus of Arf as shown by pull-down experiments" should be supported by a biochemical validation of the correct folding of both fragments, since access to information concerning how data were generated is crucial to judge their reliability. In practice, such metadata are often completely missing, with the paradox of having accuracy in down-stream experiments (annotated metadata for protein-protein interaction experiments), but no information about functionality of the molecules involved in the experiments that yielded the initial observation. It is somehow astonishing that every kind of control is requested for evaluating an experiment, except for the quality of the proteins involved. Therefore, manuscripts should be accompanied by a Minimal Information, organized in a standard format, both for evaluating protein structure and functionality, and for easy retrieval of data from different experiments/publications to use for systematic bioanalysis. In conclusion, a platform would be developed that is concerned not only with optimization of data sharing, but also with how material is controlled and data are generated. The mandatory control experiments and standardized annotation of data concerning protein functionality may be considered limiting the freedom of the scientific activity. However, MI annotation does not interfere with the scientific work, but only provides information to judge the reliability of produced results and the possibility of unambiguous data evaluation remains the backbone of scientific practice. It can be expected that improved data transparency will also increase the public acceptance for research funding.

In contrast to well defined scientific communities as, for instance, those operating in proteomics or microarrays, there is no already established organization exclusively devoted to protein production that could promote a platform like MIPFE. However, the subject will be discussed in the forthcoming Recombinant Protein Production congress (see Availability and requirements section for URL) organized by the Microbial Physiology section of the European Federation of Biotechnology and I expect to contribute to the effort with the observations reported in this commentary. The hope is that all together this information and discussions might catalyze the interest of several actors to establish a core community for the development of the MIPFE platform.

## Availability and requirements

MIBBI: 

Human Proteinpedia: 

OBI: 

FuGE: 

Addgene: 

Recombinant Protein Production congress: 

## Competing interests

The author declares that they have no competing interests.
